# Intratumoral microbiota: synergistic reshaping of lung cancer microenvironment via inflammation and immunity

**DOI:** 10.3389/fimmu.2025.1653727

**Published:** 2026-01-14

**Authors:** Ruilin Zhang, Zhao Li, Xingfei Liu, Zhengzhou Qiu, Yongxuan Li, Chenggen Gao, Changying Guo

**Affiliations:** 1 Department of Thoracic Surgery, Jiangxi Cancer Hospital, The Second Affiliated Hospital of Nanchang Medical College, Jiangxi Cancer Institute, Nanchang, China; 2 Nanchang University Jiangxi Medical College, Nanchang, China; 3 Suzhou BOE Hospital, Suzhou, China; 4 Jiangxi Key Laboratory of Oncology, Jiangxi Cancer Hospital, Nanchang, China

**Keywords:** biomarker, immunotherapy, intratumoral microbiota, lung cancer, TME

## Abstract

As high-throughput sequencing tools have advanced in recent years, scientists have discovered that lung cancer tissues are not sterile. The intratumoral microbiota exists in the tumor parenchyma and stroma in a low-biomass form. This finding has overturned the traditional concept of “sterile tumors” and brought the intratumoral microbiota to the forefront of tumor research. In this review, we focus on elucidating the mechanisms by which intratumoral microbiota influence lung cancer cells and the tumor microenvironment (TME), with the aim of clarifying their role in lung cancer progression. The intratumoral microbiota does not exist as a passive resident. Instead, it may actively induce and maintain a chronic inflammatory state through the secretion of metabolites, activation of signaling pathways, immune suppressor cell recruitment, and upregulation of immune checkpoint molecule expression, thereby promoting tumor cell proliferation, invasion, and immune evasion. From a clinical translation perspective, we explore the potential of using intratumoral microbiota characteristics to predict immunotherapy efficacy. Additionally, we assess the application prospects of engineered bacteria and targeted nanobiotics, which are based on synthetic biology, in reshaping the immune microenvironment. However, the field still faces significant challenges, particularly as the low biomass nature of lung tissues makes sequencing data highly susceptible to reagent contamination and batch effects. Additionally, the synergistic role of non-bacterial components such as fungi and viruses in the tumor ecosystem is often overlooked. Future research needs to establish rigorous quality control standards and integrate multi-omics technologies to comprehensively analyze the dynamic interaction network between the microbiota and host immunity, which will drive the clinical implementation of microbiome-based precision diagnostic and therapeutic strategies for lung cancer.

## 1 Introduction

Lung cancer is one of the most common malignant tumors with high incidence and mortality rates globally, posing a significant threat to human health (1). Despite significant recent advances in targeted and immunotherapy, the therapeutic outcomes for lung cancer are still limited due to the complexity of the TME and the lack of reliable biomarkers (2). With the continuous progress of analytical tools and detection technologies such as quantitative PCR, immunohistochemistry, 16S rRNA sequencing, and multi-omics techniques, scientists have discovered that solid tumors like colorectal, lung, and breast cancer contain microbial populations (3, 4).

In normal lung tissues, the balance of the pulmonary microbiota is maintained by phyla such as Firmicutes, Bacteroidetes, and Proteobacteria (5). However, the occurrence of lung cancer disrupts this equilibrium, leading to significant alterations in the microbial community structure. The lung is rich in mucosal tissues and is directly exposed to the external environment, which provides conditions for microbial colonization (6). The hypoxic, immunosuppressive, and nutrient-rich TME serves as an ideal habitat for intratumoral microbiota (7). Intratumoral microbiota and their metabolites are believed to potentially contribute to the formation of a specific inflammatory and immune microenvironment in lung cancer by inducing local inflammation at colonization sites and modulating immune responses (5, 8, 9). Furthermore, intratumoral microorganisms may enhance the proliferative, apoptotic, invasive, and metastatic capacities of lung cancer cells through various complex regulatory mechanisms, and could even exert a potential influence on treatment efficacy (10, 11). Therefore, a deeper investigation into the intricate relationship between intratumoral microbiota and the lung cancer TME not only contributes to elucidating the pathogenesis of the disease, but may also offer critical clues for developing novel diagnostic markers, prognostic indicators, and therapeutic strategies. This holds promise for improving the long-term prognosis of patients with lung cancer.

## 2 Sources and characteristics of intratumoral microbiota in lung cancer

### 2.1 Sources of intratumoral microbiota in lung cancer

Firstly, mucosal barrier invasion. The respiratory mucosal barrier acts as the host’s first line of defense against exogenous pathogenic factors (12, 13). During dysbiosis, compromised intercellular adhesion and increased barrier permeability lead to barrier dysfunction. This creates conditions conducive to ectopic microbial colonization, fostering a carcinogenic inflammatory milieu. Furthermore, tumors themselves can inflict damage upon the mucosal barrier. For example, lung cancer cells carrying TP53 gene mutations can impair the pulmonary epithelial barrier, leading to bacterial infiltration. This results in the establishment of a distinct bacterial community within lung tumors, dominated notably by the genus *Acidovorax* (14, 15).

Secondly, adjacent tissue invasion. Given the high degree of similarity between the microbial communities of tumor tissue and normal adjacent tissue (NAT), many studies propose NAT as a potential reservoir for the intratumoral microbiota (7, 13, 16). Spatial meta-transcriptomics has revealed a gradient distribution of bacterial burden within the lung cancer microenvironment: the highest bacterial load is detected in the respiratory tract lumen, followed by progressively lower levels in NAT and a further reduction within lung tumor cells. This dynamic spatial pattern suggests that microbes colonizing the tumor may migrate from the lower respiratory tract, utilizing NAT as a biological conduit, via mucosal barrier invasion (17).

Finally, hematogenous invasion. Microorganisms from the oral cavity, gut, respiratory system, and other possible locations can translocate through the circulation to colonize tumors (18). From a pathophysiological perspective, the breach of mucosal barrier integrity represents the initial step triggering microbial hematogenous dissemination (19). When the respiratory or gastrointestinal mucosal barrier is compromised, resident microbiota can enter the circulation through inflamed vasculature and traffic to the abnormally enriched vascular network within tumor tissue (20, 21). Studies demonstrate that upon oral gavage of *Akkermansia muciniphila (Akk*) in murine lung cancer models, the abundance of this bacterium significantly increases within tumor tissue (22). Crucially, concurrent detection via 16S rDNA sequencing revealed a substantial rise in *Akk* levels in blood samples just 2 hours post-administration. As for the oral microbiota, the link between the oral cavity and the respiratory and gastrointestinal tracts may offer a new opportunity for lung cancer development (23, 24). Meanwhile, a study on periodontitis and cancer shows that, compared to individuals with healthy periodontal tissues, periodontitis patients have an increased relative risk of lung cancer and higher mortality (25). However, research indicates that the majority of tumor-associated bacteria and fungi exist inside cells, within both tumor cells and immune cells. This intracellular localization suggests that microbes likely migrate into tumors or adjacent tissues as fragments or intact cells, rather than existing in a free-floating state (26). Supporting this paradigm, studies confirm blood of healthy individuals contains microorganisms, with higher concentrations of bacterial DNA found within red blood cells (RBCs) than in plasma (27). For example, viable bacteria including *Streptococcus pneumoniae* and *Staphylococcus aureus* have been detected inside RBCs in some contexts (28). Collectively, these findings imply a potential mechanism whereby microbes may utilize RBCs as “carriers.” Guided by chemotactic gradients emanating from necrotic tumor debris, these microbe-laden RBCs could then infiltrate tumor tissue through compromised vasculature (7).

**Figure 1:**
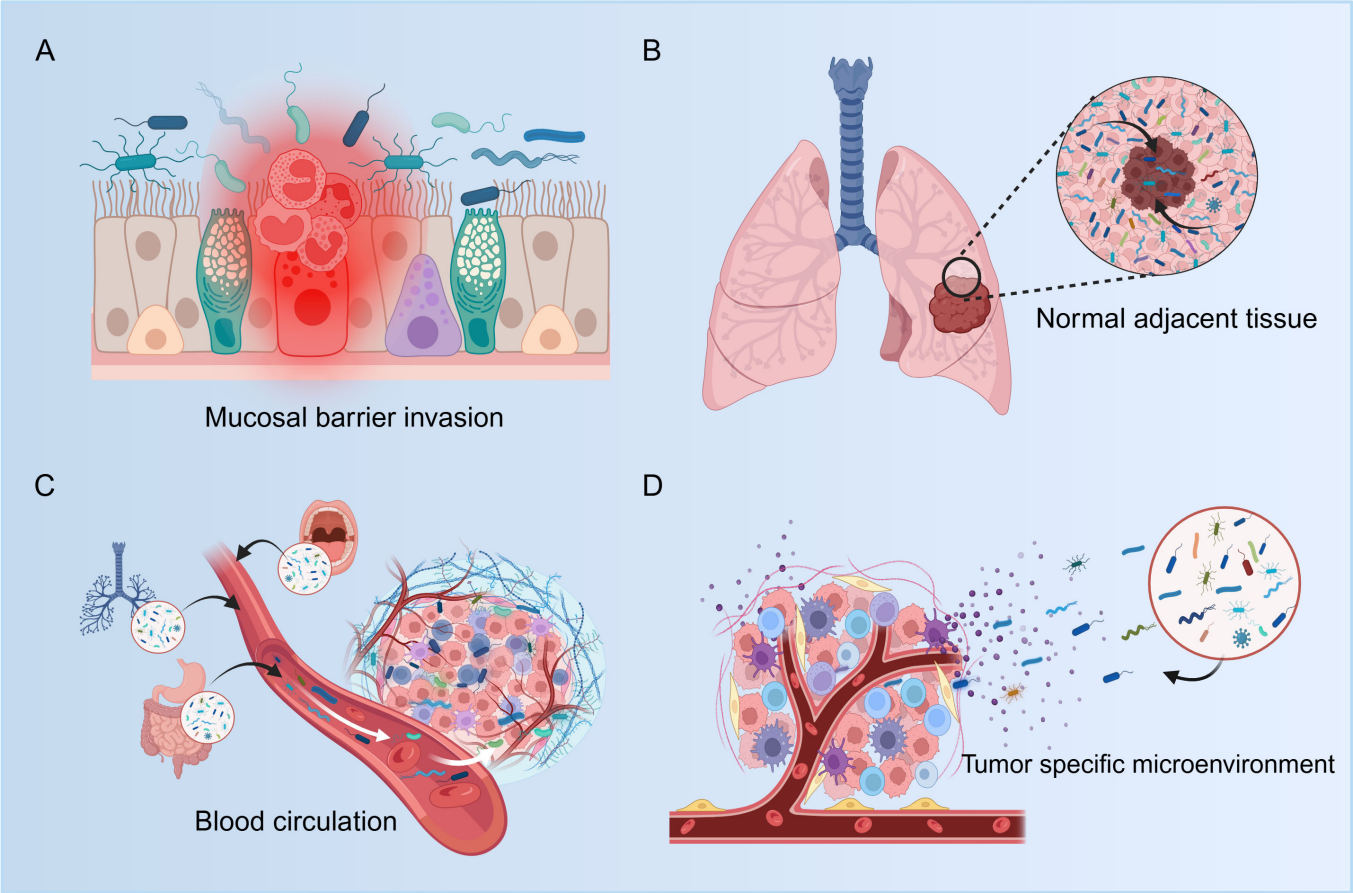
The sources of intratumoral microbiota in lung cancer. **(A)** Mucosal barrier invasion. **(B)** Adjacent tissue invasion. **(C)** Hematogenic invasion. **(D)** Tumor specific microenvironment. Created in BioRender (2025). https://BioRender.com/z007obt.

In addition, specific features of the TME may actively promote microbial colonization within tumors (29). First, the characteristically hypoxic nature of the TME creates a favorable niche for facultative and anaerobic bacteria to survive and proliferate (30). Second, the hyperpermeability of endothelial cells during tumor angiogenesis provides opportunities for microbiota within the peripheral circulation to extravasate across the vascular wall and enter the tumor parenchyma. Moreover, components derived from dying tumor cells within the tumor mass can serve as abundant nutrient sources for bacterial growth (31). Notably, tumor cells themselves may secrete chemoattractant compounds capable of recruiting bacteria (28). Critically, the TME typically exhibits an immunosuppressive state, which effectively shields bacteria from immune clearance (32) (**Figure 1**).

### 2.2 The heterogeneity of intratumoral microbiota distribution in lung cancer

The microbial signals detected within lung cancer tumors are not randomly distributed; rather, their community characteristics exhibit high heterogeneity. This differential distribution of the microbial landscape is closely associated with tumor histological subtypes, tumor progression stages, and patient demographic characteristics, suggesting that intratumoral microorganisms may have potential intrinsic connections with specific tumor biological behaviors.

Lung cancers with different pathological subtypes exhibit distinct differences in the composition of their intratumoral microbiota. Specifically, *Acidovorax*, *Klebsiella*, and *Anaerococcus* are found in much higher abundance in lung squamous cell carcinoma (LUSC) (14). Conversely, lung adenocarcinoma (LUAD) shows higher DNA signal abundance of *Acinetobacter, Brevundimonas, Propionibacterium, Cyanobacteria*, and *Thermus* (33). The distribution of intratumoral microbiota is associated with tumor progression stages. In advanced tumors, the abundance of genera such as *Thermus* and *Corynebacterium* is significantly higher than in stage I-II tumors. Additionally, *Legionell*a is more prevalent in patients with metastasis (32). Moreover, the composition of the lung cancer microbiota exhibits age-and sex-related variations. In LUSC, the abundance of *Pseudomonas putida str. KT2440* is significantly higher in tumors from younger male patients. In LUAD, tumors from older patients, regardless of sex, show a higher abundance of *Escherichia coli str. K-12 substr. W3110* (34).

## 3 Effects of the intratumoral microbiota on lung cancer cells

### 3.1 Genomic instability and DNA damage

Intratumoral microbiota and their toxins can induce genomic instability and DNA damage in host cells, a key early event driving tumorigenesis. While certain bacterial strains are known to secrete directly genotoxic metabolites—a mechanism well-established in colorectal cancer—their role in lung cancer remains exploratory. A canonical example is pks+ *Escherichia coli*, which produces colibactin, an α-aminoketone DNA alkylating agent (35, 36). Colibactin preferentially targets adenine-rich DNA regions, inducing interstrand crosslinks and double-strand breaks (37, 38). This severe DNA damage activates the ATM–Chk2 signaling pathway, leading to dysregulated cell cycle control, cytotoxicity, and mutation accumulation, thereby promoting tumor initiation (39, 40). Beyond *E. coli*, Gram-negative *Campylobacter jejuni* secretes the cytolethal distending toxin (CDT), which exhibits DNase activity. CDT can directly degrade double-stranded DNA or cause DNA alkylation, compromising genomic stability, typically through direct bacterium–host cell contact (18). In addition to the direct physical damage caused by bacterial toxins, oncogenic viruses disrupt genome integrity by interfering with cell cycle checkpoint proteins. Human papillomavirus (HPV) has been detected in non-small cell lung cancer (NSCLC) (41). High-risk HPV subtypes, such as HPV16 and HPV18, express the oncogenic proteins E6 and E7, which degrade p53 and inactivate Rb proteins, respectively (42, 43). This leads to the failure of cell cycle regulation and the accumulation of genomic instability.

Microbial metabolites can also compromise DNA integrity via chemical carcinogenesis. Aflatoxin B1 (AFB1), a secondary metabolite produced by *Aspergillus flavus*, is metabolically activated in the lung by cytochrome P450 enzymes to form AFB1-exo-8,9-epoxide (AFBO) (44). This reactive compound covalently binds to guanine residues in DNA, forming DNA adducts that induce mutations and contribute to lung carcinogenesis (45).

Furthermore, microbiota can indirectly affect genomic integrity by modulating oxidative stress and post−transcriptional regulation, as demonstrated in various lung infection models (46). For instance, *Bacteroides fragilis toxin* (Bft) induces reactive oxygen species (ROS) accumulation, leading to oxidative DNA damage (47). At the post-transcriptional level, *Mycoplasma* infection destabilizes RNA and upregulates bone morphogenetic protein 2 (BMP2) expression, thereby enhancing the malignant phenotype of lung adenocarcinoma cells (48). Although these molecular mechanisms of microbiota-induced genomic instability have been validated in cellular models, their specific contributions to human lung carcinogenesis require further validation through clinical multi-omics studies.

### 3.2 Epigenetic regulation

Intratumoral microbiota profoundly influences lung cancer progression by altering the epigenetic landscape of tumor cells, specifically through modulating the expression of genes involved in malignant transformation. This aberrant epigenetic modification primarily drives tumorigenesis and progression by silencing tumor suppressor genes (TSGs) or activating proto-oncogenes (49).

At the level of DNA methylation, intratumoral microbes can reshape the genome by regulating methyltransferase activity or substrate availability. Studies have shown that the abundance of *Fusobacterium nucleatum* (*F. nucleatum*) positively correlates with hypermethylation of the CpG island in the promoter region of the tumor suppressor gene CDKN2A (50). This suggests microbial involvement in tumor immune evasion via epigenetic silencing mechanisms. Further mechanistic research indicates a synergistic effect between *F. nucleatum* and *Hungatella hathewayi*. Together, they induce aberrant activation of DNA methyltransferases (DNMTs), leading to hypermethylation of promoter regions in multiple TSGs and their subsequent transcriptional silencing (51). While *F. nucleatum*-induced epigenetic silencing has been extensively reported in gastrointestinal cancers, its role in lung cancer appears more correlative. Although *F. nucleatum* has been detected in bronchoalveolar lavage fluid (BALF) from lung cancer patients, the specific mechanisms by which it influences lung cancer development remain unclear. Furthermore, recent research indicates that specific microorganisms detected in the lung cancer microenvironment, such as *Alphaproteobacteria* and *Deinococcus*, are enriched for the methionine synthesis pathway. This may position S-adenosylmethionine (SAM) as a key methyl donor for nucleic acid and protein methylation, thereby indirectly influencing the epigenetic modification processes in lung cancer cells (52).

Beyond DNA methylation, microbes may also exert epigenetic control through histone modifications. A mechanistic study on lung adenocarcinoma found that *Stenotrophomonas maltophilia* can upregulate the expression of histone deacetylase 5 (HDAC5) gene, inducing aberrant activation of the Apelin signaling pathway and thereby enhancing tumor cell migration (53). However, it is important to note that this study utilized formalin-fixed, paraffin-embedded lung tumor samples rather than fresh tissues. The research did not rule out potential alterations in HDAC5 gene expression results due to DNA damage possibly caused by formalin fixation. Microbial metabolites can also interfere with histone modifications as substrates or inhibitors. For instance, butyrate, produced by the intratumoral commensal bacterium *Roseburia*, can significantly increase acetylation levels at the H3K27 site by inhibiting the binding of histone deacetylase HDAC2 to the H19 promoter (54). This activates the transcriptional activity of the proto-oncogene H19, whose aberrant expression is closely associated with lung cancer cell proliferation and epithelial-mesenchymal transition (EMT) (55, 56). Notably, the role of butyrate is subject to debate. While some studies propose it is beneficial for health and possesses tumor-suppressive properties in the intestinal context, its effects in the TME, particularly in lung cancer, may be complex and context-dependent (57).

### 3.3 Metabolite regulation and metabolic reprogramming

Intratumoral microbiota contribute to enhanced invasiveness and environmental adaptability of lung cancer cells by secreting bioactive metabolites and modulating the energy metabolism pathways of tumor cells.

Microbiota-derived short-chain fatty acids (SCFAs) can exhibit tumor-promoting properties in specific contexts. Propionate, a product of microbial metabolism, can be metabolically obstructed within cancer cells due to downregulated expression of the key enzyme methylmalonyl-CoA epimerase (MCEE) (58). This obstruction occurs under the influence of a metastasis signal-driven ERK2-mediated Sp1/EGR1 transcriptional switch. It leads to abnormal intracellular accumulation of methylmalonic acid (MMA), which significantly enhances lung cancer cell invasiveness (58). Consequently, targeting key nodes in propionate metabolism (such as the MMA-generating pathway) is considered a potential therapeutic strategy for intervening in metastatic cancer (59). Beyond SCFAs, microbial tryptophan catabolites (e.g., indoles) are also implicated in influencing lung cancer progression. These metabolites can promote the nuclear translocation of the aryl hydrocarbon receptor (AhR) and the transcription of its target genes. By inducing regulatory T cell (Treg) differentiation and tolerogenic polarization of macrophages, they suppress the anti-tumor activity of inflammatory T cells, thereby reshaping the immune microenvironment (60). Furthermore, recent studies have revealed a dynamic compensatory relationship between indoleamine 2,3-dioxygenase 1 (IDO1), a key enzyme in the tryptophan metabolism pathway, and the immune defense molecule guanylate-binding protein 1 (GBP1). Loss of IDO1 can compensatorily upregulate GBP1 and inducible nitric oxide synthase (iNOS) thereby maintaining host defense responses against intracellular microbes (61). This finding further underscores the complex, bidirectional mechanisms within the tryptophan metabolic network in regulating immune homeostasis in the microenvironment.

Intratumoral microbiota can also regulate tumor cell energy metabolism by modulating glycolysis and glutaminolysis. Tumor cells often gain a developmental advantage through metabolic reprogramming (e.g., the Warburg effect), utilizing lactate, glutamine metabolites (glutamate, succinate), and nucleotide precursors (62). For instance, tumor cells exacerbate the reprogramming of glucose or glutamine metabolism, driving excessive secretion and accumulation of lactate. This lactate accumulation not only restricts glucose uptake by effector T cells but also directly suppresses the cytotoxic function of CD8^+^ T cells and promotes Treg generation, thereby facilitating tumor immune escape (62). Additionally, lactate can act as a signaling molecule. By activating hypoxia-inducible factor-1 (HIF-1) and its downstream vascular endothelial growth factor receptor (VEGFR) in endothelial cells, lactate helps maintain a hypoxic and pro-angiogenic microenvironment (63). However, this metabolic reprogramming exhibits bidirectional plasticity. Intriguingly, research has found that the gut-derived bacterium *Akk*, after migrating to lung tumor tissue via the bloodstream, can reprogram glycolysis and glutamine metabolism pathways. This activity suppresses the expression of lactate dehydrogenase A (LDHA) and lactate accumulation within the TME, thereby reversing the aforementioned tumor-promoting metabolic state (22). This indicates that not all intratumoral microbes promote immunosuppression; different microbial communities exert specific and potentially opposing functions in remodeling the TME.

### 3.4 Activation of oncogenic pathways and promotion of metastasis

Intratumoral microbiota may promote tumor cell proliferation, invasion, and metastasis by activating key pro-oncogenic signaling pathways within cancer cells.

Specific respiratory tract microbiota are closely associated with the aberrant activation of classic oncogenic pathways. DNA signatures of oral-derived bacterial taxa (e.g., *Streptococcus* and *Veillonella*) were detected at significantly higher relative abundances in the lower respiratory tract of lung cancer patients compared to controls. *In vitro* experiments confirm that exposure to these bacterial communities significantly activates intracellular PI3K and ERK signaling pathways in airway epithelial cells (64). Beyond kinase cascades, the Wnt/β-catenin pathway is also modulated by intratumoral microbes. Gene expression analyses reveal that the expression levels of genes involved in the Wnt/β-catenin (CTNNB1), hypoxia (HIF1A), and angiogenesis (VEGFA) pathways strongly positively correlate with bacterial load within tumors (17). Recent studies have detected the presence of Epstein-Barr virus (EBV) in lung cancer tissues (65). The EBV-encoded latent membrane protein 1 (LMP1) can mimic CD40 receptor signaling, leading to constitutive activation of the JAK/STAT and MAPK pathways. Additionally, the EBV-encoded nuclear antigen 1 (EBNA1) can interfere with the p53 pathway. This persistent signal input directly drives abnormal proliferation of tumor cells (66). However, given the prevalence of latent EBV infection in the human population, establishing a definitive causal relationship between EBV and lung cancer pathogenesis remains challenging.

Intratumoral microbiota can also activate specific pro-oncogenic signaling axes by inducing inflammatory mediators. The genus *Peptococcus* has been identified as an independent prognostic factor for poor outcomes in NSCLC, with its oncogenic mechanism primarily relying on the activation of the tumor necrosis factor (TNF) signaling pathway (67). Further studies show a significant positive correlation between the abundance of *Peptococcus* and the expression of key TNF pathway components (CXCL1, PTGS2, and IRF1) (67). Research in colorectal cancer has found that *Peptococcus* activates NF‐κB to promote CXCL1 secretion. CXCL1, in turn, recruits myeloid-derived suppressor cells (MDSCs) and inhibits the infiltration of functional T cells, thereby fostering an immunosuppressive microenvironment (68). Overexpression of PTGS2 facilitates the conversion of arachidonic acid to prostaglandin E2 (PGE2), which directly promotes tumor cell proliferation (69). These findings suggest that *Peptococcus* may remodel the inflammatory and immune microenvironment via a CXCL1-PTGS2-IRF1 axis, ultimately leading to worse patient prognosis. The precise relevance of this mechanism in lung cancer warrants further in-depth investigation.

In addition to activating oncogenic pathways, intratumoral microbiota may promote lung cancer metastasis by inducing epithelial-mesenchymal transition (EMT). For instance, *F. nucleatum* and *Bacteroides fragilis* can enhance the adhesion of lung cancer cells to endothelial cells, conferring invasive properties that facilitate extravasation into the bloodstream and subsequent dissemination to distant organs via circulation (70). The EMT process can be further driven by activating signaling pathways such as PI3K/AKT, Wnt/β-catenin, TGFβ/SMAD, and MAPKs, which propel cancer cell invasion and spread (71). Recent studies also indicate that intratumoral microbes can promote lung cancer metastasis by upregulating matrix metalloproteinases (MMPs) to induce extracellular matrix (ECM) remodeling and by weakening immune surveillance (72, 73).

**Figure 2:**
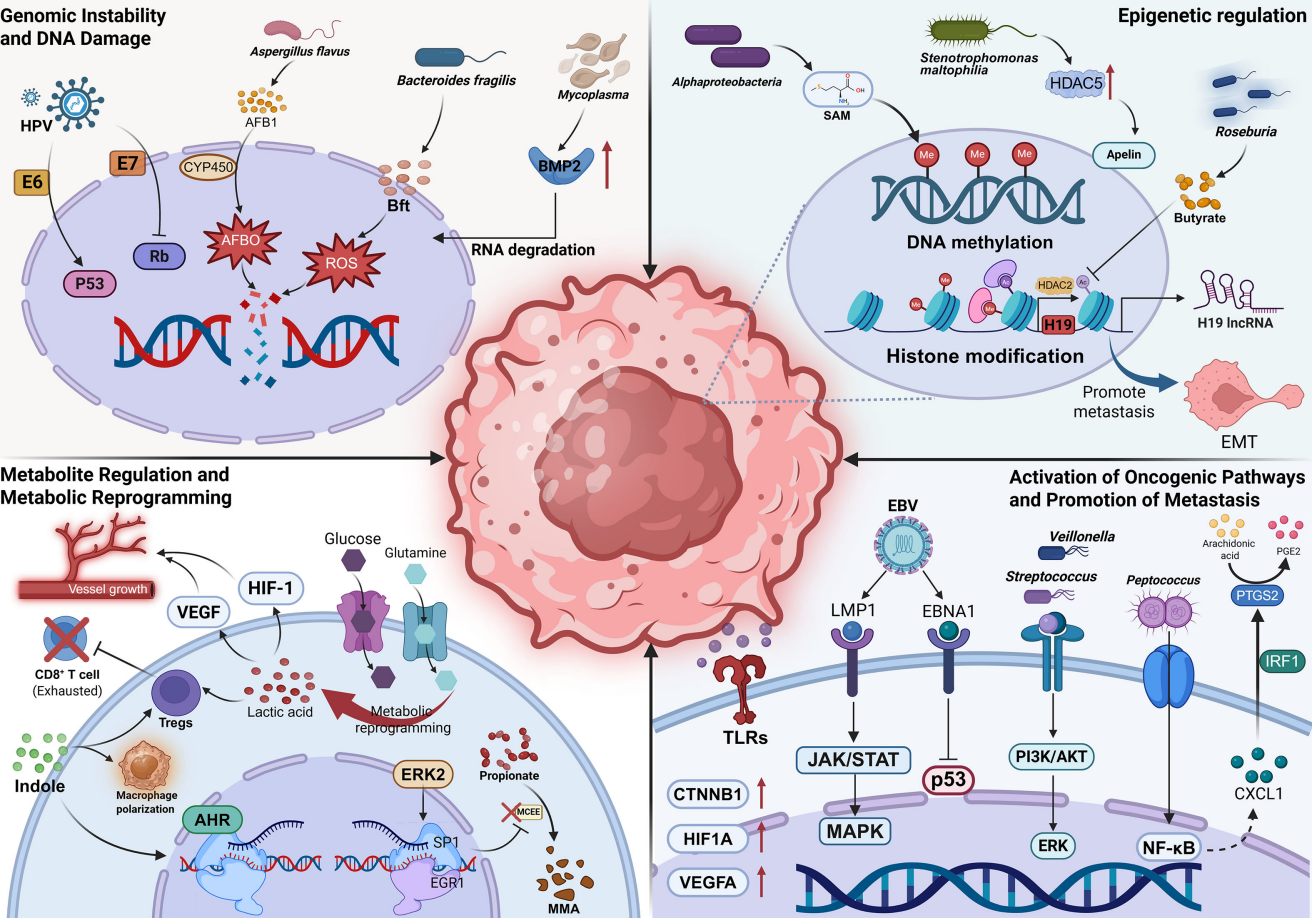
The influence of intratumoral microbiota on lung cancer cells. Created in BioRender (2025). https://BioRender.com/yyfwalc.

Collectively, the above mechanisms demonstrate that the influence of intratumoral microbiota on lung cancer cells is not mediated through a single, isolated pathway but rather through the interplay and synergy of multiple mechanisms (**Figure 2**). They directly enhance malignant phenotypes by inducing genotoxicity-mediated genomic instability, regulating epigenetic remodeling, and driving metabolic reprogramming. Crucially, these cell-intrinsic alterations do not occur in isolation. The resulting metabolic byproducts and inflammatory signals extend beyond the tumor cells themselves, laying the groundwork for the remodeling of the TME.

## 4 Effects of intratumoral microbiota on the TME in lung cancer

### 4.1 Inflammatory microenvironment

Intratumoral microbiota induce and sustain a chronic, non-specific inflammatory state by persistently activating the host’s innate immune signaling. This tumor-promoting inflammation constitutes a crucial breeding ground for the initiation and progression of lung cancer. Existing immunological evidence clearly indicates that intratumoral microbiota are not only components of the microenvironment but also potential stimuli that trigger immune responses (74, 75). They interact with the host immune system via specific molecular patterns, profoundly reshaping the inflammatory landscape within the tumor. A core mechanism in this process is the sustained activation of pattern recognition receptors (PRRs), particularly Toll-like receptors (TLRs). For instance, in patients with chronic obstructive pulmonary disease (COPD), an increase or dysbiosis of non-typeable Haemophilus influenzae (NTHi) in the lungs allows bacterial antigenic components, such as lipopolysaccharide (LPS), to be recognized by TLRs (76). This recognition subsequently activates downstream NF-κB and STAT3 signaling pathways, inducing the generation and activation of Th17 cells. NF-κB, as a key transcription factor for inflammatory responses, directly drives the transcription and release of pro-inflammatory cytokines (e.g., IL-17C) upon activation, thereby helping to maintain an inflammatory state in the local microenvironment. Importantly, NTHi not only directly stimulates epithelial cells to release IL-17C but can also enhance the expression of neutrophil chemotactic factors (KC and MIP-2) via IL-17C, leading to massive neutrophil recruitment and the establishment of a tumor-promoting inflammatory microenvironment (77). This pathogen-driven inflammatory response is not exclusive to bacteria. Parasites like *Trichomonas tenax* can also induce the sustained secretion of pro-inflammatory factors such as IL-6, maintaining a low-grade chronic inflammatory state even without causing epithelial damage (78). A more aggressive mechanism of inflammatory remodeling is seen in SARS-CoV-2 infection, where the triggered high-level IL-6 cytokine storm not only exacerbates tissue damage but has also been found to awaken dormant disseminated cancer cells (DCCs) in the lungs (79).

Within this context of chronic inflammation, specific microbe-cytokine axes contribute to the formation of the inflammatory microenvironment. Studies have confirmed that various intratumoral commensal bacteria, including *Staphylococcus*, *Streptococcus*, *Lactobacillus*, and *Pasteurellaceae*, can stimulate alveolar macrophages to secrete Myd88-dependent IL-1β and IL-23 (80). This, in turn, induces the proliferation and activation of Vγ6Vδ1 γδ T cells, leading to the sustained production of effector molecules IL-17 and IL-22 (80). Notably, tumor-associated macrophages (TAMs) exhibit high plasticity. Microbial stimulation can induce M1-type macrophages to release ROS, NO, and pro-inflammatory cytokines (such as TNF-α and IL-6) to initiate inflammatory responses (81). Concurrently, the dynamic conversion and co-existence of M1 and M2 phenotypes further orchestrate a complex cytokine network. This macrophage-dominated inflammatory cascade provides the essential microenvironmental support for tumor cell survival and proliferation (82). This process ultimately forms a characteristic inflammatory microenvironment dominated by IL-17 and IL-22, which directly promotes the malignant transformation of lung cancer cells. Furthermore, the same study found that in this mouse lung cancer model, γδ T cells highly express the EGFR ligand amphiregulin (Areg) at both the mRNA and protein levels (80). Areg can act as a key molecular node linking inflammation, tumor growth, and therapy resistance. High levels of Areg can persistently activate EGFR downstream RAS-MAPK and PI3K-AKT signaling pathways, driving tumor cell proliferation (83). Moreover, under EGFR tyrosine kinase inhibitor (TKI) treatment, Areg overexpression can serve as a bypass activation mechanism to maintain tumor cell survival, thereby leading to drug resistance. On the other hand, Areg can promote the expansion of Tregs and the release of inhibitory cytokines (such as IL-10), thereby weakening the cytotoxic efficacy of T cells and contributing to resistance against immunotherapies (e.g., PD-1/PD-L1 inhibitors) (84).

In addition to bacterial surface antigens, toxins secreted by microbes may also be important drivers of inflammatory storms. Metagenomic analysis of NSCLC samples revealed a markedly elevated relative abundance of DNA sequences assigned to Cyanobacteria capable of producing microcystins (MCs) (85). Mechanistic studies indicate that the presence of MCs is closely associated with elevated activity of poly(ADP-ribose) polymerase 1 (PARP1). The hyperactivation of PARP1 triggers the release of a series of pro-inflammatory cytokines (such as TNF-α, IL-1β, IL-4), thereby exacerbating the formation of a chronic inflammatory environment (86). However, this study had a relatively small clinical sample size and was primarily based on correlative analyses. It remains difficult to definitively determine whether microcystins are an initiating factor in lung cancer induction or a secondary product resulting from alterations in the TME.

**Figure 3:**
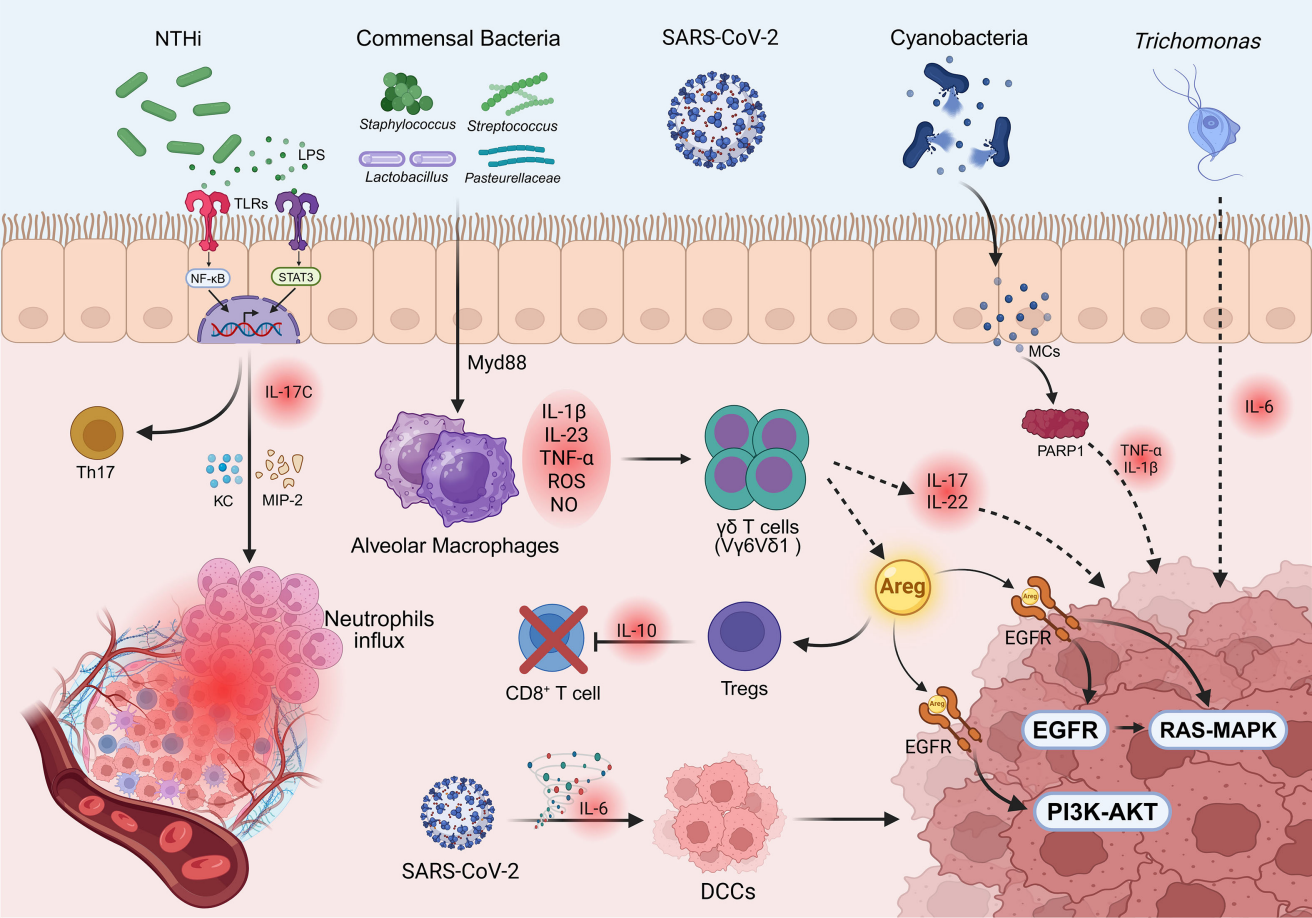
The influence of intratumoral microbiota on the inflammatory microenvironment of lung cancer. Created in BioRender (2025). https://BioRender.com/u1apxrg.

These persistently present inflammatory mediators not only directly damage tissue but also recruit immune cells, laying the foundation for the subsequent remodeling of the immunosuppressive microenvironment (**Figure 3**).

### 4.2 Immune microenvironment

Following the establishment of tumor-promoting inflammation, intratumoral microbes can further contribute to the construction of an immunosuppressive TME by recruiting immunosuppressive cell populations. The intratumoral microbiota often triggers immune tolerance programs through the activation of pattern recognition receptors (PRRs), leading to a decreased proportion of CD8^+^ T cells and an increased proportion of Tregs among tumor-infiltrating lymphocytes. This phenomenon has been observed in lung, pancreatic, and colorectal cancers (87). For instance, *Veillonella parvula*-induced pulmonary dysbiosis can trigger Th17 cell recruitment, accompanied by reduced CD4^+^ T cell infiltration, elevated IL-17 secretion, an increased proportion of PD-1^+^ T cells, and neutrophil infiltration, thereby weakening anti-tumor immune surveillance (88). Recent studies highlight that the role of fungi in lung cancer progression should not be overlooked. *Aspergillus sydowii* detected in lung tumors secretes IL-1β via the β-glucan/Dectin-1/CARD9 pathway, triggering the expansion and activation of myeloid-derived suppressor cells (MDSCs), which in turn impair cytotoxic T cell activity and lead to the accumulation of PD-1^+^ CD8^+^ T cells (89).

Microbial metabolites can also reshape the functional phenotypes of immune cells. On one hand, metabolites can regulate macrophage polarization. For example, butyrate derived from *Roseburia*, while inhibiting inflammatory T cell infiltration, can also promote macrophage polarization towards an M2 phenotype and induce the secretion of inhibitory factors such as IL-10, IL-13, and macrophage inflammatory protein 3α (MIP-3α), thereby establishing an immunosuppressive barrier (54). On the other hand, short-chain fatty acids (SCFAs) produced by anaerobic bacteria in the lower respiratory tract have been found to hinder IFN-γ production by CD4^+^ and CD8^+^ T cells, directly inducing effector T cell exhaustion (90). This microbiota-driven immune cell imbalance was further corroborated in a mouse lung cancer model, where the abundance of intratumoral *Coriobacteriaceae* was positively correlated with M2 macrophages and negatively correlated with CD8^+^ T cells, suggesting this bacterial family directly contributes to immunosuppressive microenvironment formation. Conversely, the intratumoral abundance of *Pasteurella* was positively correlated with CD8^+^ TILs and negatively correlated with M2 macrophages (91). This stark contrast in immune signatures underscores that not all intratumoral microbes promote immunosuppression; different microbes exert specific and divergent functions in shaping the tumor immune landscape.

Intratumoral microbes may also block immune-killing pathways by secreting virulence factors or modulating immune checkpoints. Research has found that protein tyrosine phosphatase A (PtpA), a key virulence factor secreted by *Mycobacterium tuberculosis*, can dephosphorylate signaling proteins such as p-JNK, p-p38, and p-VPS33B in the cytoplasm to suppress innate immune responses (92). Additionally, within the host cell nucleus, PtpA participates in regulating the expression of genes like GADD45A to promote the proliferation and migration of lung adenocarcinoma cells. Furthermore, intratumoral microbes can influence the immune microenvironment by regulating checkpoint proteins. Elevated levels of various immune checkpoint molecules—including programmed cell death protein 1 (PD-1), cytotoxic T-lymphocyte-associated protein 4 (CTLA-4), lymphocyte-activation gene 3 (LAG-3), B- and T-lymphocyte attenuator (BTLA), and V-domain Ig suppressor of T cell activation (VISTA)—have been detected in EBV-positive lung cancer tissues (66). These immune inhibitors may promote EBV-associated tumor immune tolerance, thereby enhancing tumor survival. Another study revealed that the Fap2 protein expressed by *F. nucleatum* can directly bind to the checkpoint protein TIGIT, inhibiting the anti-tumor activity of natural killer (NK) cells and T cells (93). It should be noted, however, that this finding was not validated using lung cancer tissue. Although *F. nucleatum* has been detected in BALF from lung cancer patients, further validation in lung cancer models is required to determine whether such low-abundance bacteria in lung tumor tissue can reach the threshold necessary to trigger immune checkpoint blockade.

Intercellular communication is another crucial means by which microbes participate in reshaping the immune microenvironment, with extracellular vesicles (EVs) acting as key messengers. Emerging immunological perspectives identify that EVs are efficient delivery vehicles within the TME, facilitating complex communication between tumor cells and immune cells (94). Tumor cells colonized by microbes can secrete exosomes loaded with specific cargo, which includes not only miRNAs but also various proteins and peptides capable of altering the microenvironment (95). For example, studies in colorectal cancer have found that tumor cells infected with *F. nucleatum* release exosomes enriched with specific miRNAs (e.g., miR-1246, miR-92b-3p) and the chemokine CXCL16 (96). Through paracrine mechanisms acting on neighboring cells, these exosomes can not only target and inhibit GSK3β to activate the Wnt/β-catenin pathway, enhancing invasiveness, but have also been shown to modulate Treg cell proliferation and systemically suppress anti-tumor immune responses (97). Although the specific mechanisms in lung cancer are still under investigation, this strategy of remote immune regulation by delivering bioactive molecules via EVs is likely a common mechanism employed by intratumoral microbes to achieve immune escape.

**Figure 4:**
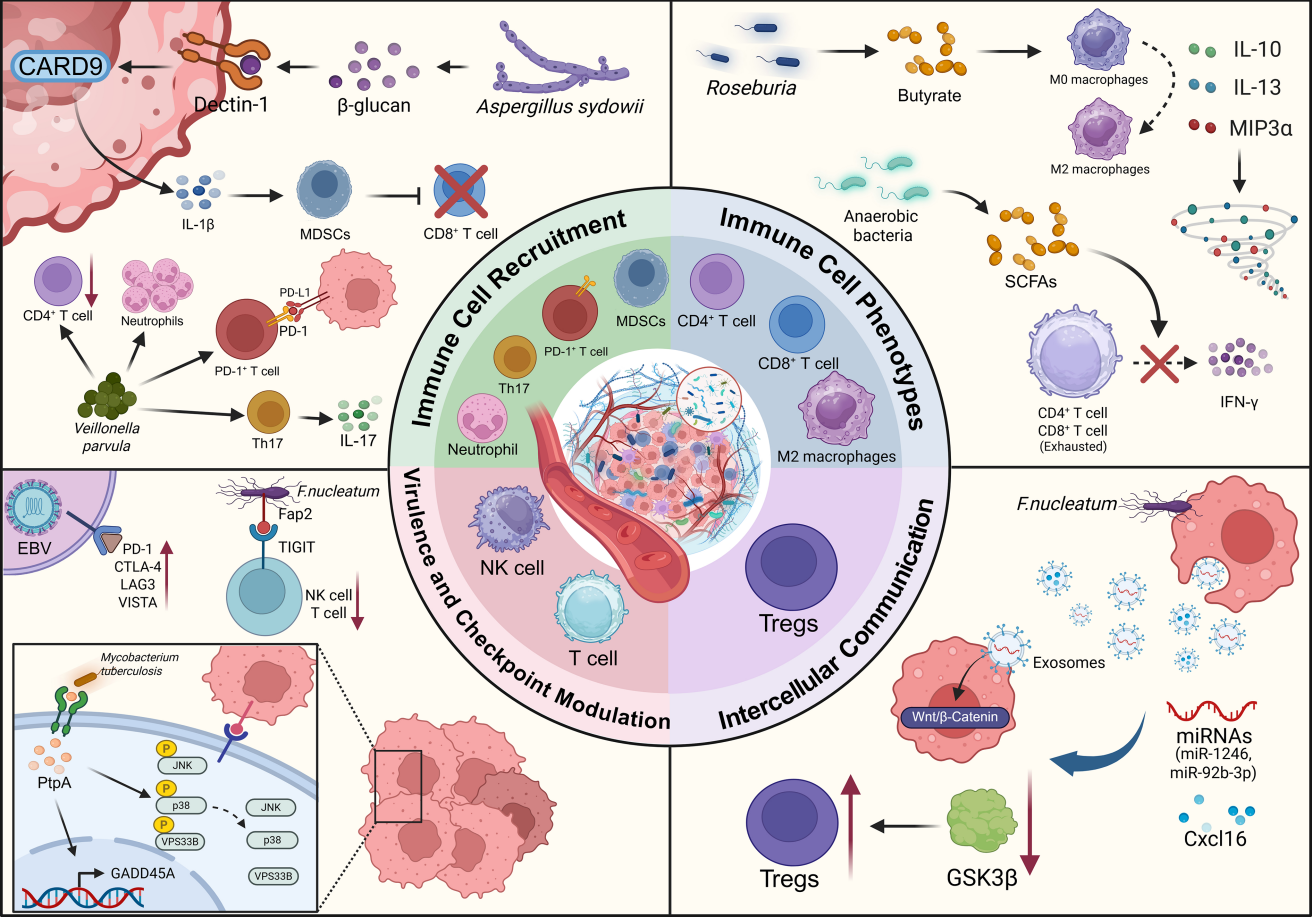
The influence of intratumoral microbiota on the immune microenvironment of lung cancer. Created in BioRender (2025). https://BioRender.com/84h0ssi.

Collectively, these mechanisms suggest that the intratumoral microbiota may be a significant factor driving the transition from a chronic inflammatory microenvironment to a state of persistent adaptive immunosuppression, thereby influencing immune escape and therapy resistance in lung cancer (**Figure 4**).

## 5 The impact of intratumoral microbiota on immunotherapy response

Immunotherapy has shown significant efficacy in clinical cancer treatment, opening new avenues for cancer management. Notably, the intratumoral microbiota is identified as an essential factor influencing immunotherapy outcomes (98, 99).As mentioned earlier, intratumoral microbiota-induced γδ T cells can highly express Areg. This not only contributes to the formation of an immunosuppressive barrier by enhancing Treg function but also directly attenuates the host’s anti-tumor immune response, thereby constituting a potential resistance mechanism to immunotherapy. In lung cancer immunotherapy, *Akk* abundance predicts patient response to immune checkpoint inhibitors (ICIs) and is related with resistance to PD-1 inhibitors (100). Notably, this bacterium has been detected in lung tumors (22). Further investigation revealed that oral administration of its immunogenic strain, Akkp226118, can restore sensitivity to PD-1 inhibitor therapy by inducing dendritic cells to secrete IL-12. This, in turn, promotes the recruitment of CCR9^+^CXCR3^+^ CD4^+^ T lymphocytes into the TME (101).

Integrative multi-omics analysis of the metastatic tumor microbiome identified that persistently high *F. nucleatum* abundance was substantially associated with shorter overall survival (OS) and progression-free survival (PFS) in patients with metastatic NSCLC. At the transcriptomic level, the TME enriched with *F. nucleatum* exhibits a pronounced immunosuppressive signature. This is characterized by the coordinated downregulation of genes encoding cytotoxic effector molecules, components of the IFN-γ response pathway, and major histocompatibility complex (MHC) class II molecules. These findings suggest that this bacterial genus may be involved in biological processes leading to impaired antigen presentation and functional silencing of T cells (102). Metagenomic sequencing analysis of BALF from lung cancer patients receiving anti-PD-1/PD-L1 immunotherapy revealed that the abundance of *F. nucleatum, Neisseria* sp*ecies*, and *Veillonella dispar* was associated with resistance to immunotherapy (103). Additionally, low PD-L1 expression and poor reactivity to checkpoint inhibitors are correlated with the presence of *Gammaproteobacteria* in bronchoscopic tumor samples of immunotherapy patients (104). These clinical evidence collectively suggests that the specific microbiome structure in the lungs may contribute to the development of a low-immunogenic tumor phenotype, potentially leading to resistance to immunotherapy.

Fortunately, studies also reveal that combining specific microbial consortia with immune checkpoint inhibitors can enhance the efficacy of cancer immunotherapy. In an independent cohort study of NSCLC patients receiving ICI monotherapy, intratumoral detection of *Escherichia* species was associated with improved OS in both univariate and multivariate analyses (105). Furthermore, transcriptomic profiling demonstrated that *Escherichia*-positive tumor samples correlated with gene expression signatures indicative of enhanced immune cell infiltration (105). Furthermore, commensal Bifidobacteria can enhance anti-tumor immunity and the efficacy of anti-PD-L1 therapy by augmenting the antigen-presenting function of dendritic cells (DCs), which triggers the accumulation of CD8^+^ T cells within the TME (106). Subsequent research revealed that Bifidobacteria can specifically accumulate in the TME. The bacterial DNA they release is recognized by the host DCs via the cGAS-STING pathway, inducing the secretion of type I interferons. This interferon-dependent paracrine signaling promotes the cross-presentation of tumor antigens by DCs, thereby enhancing the immune response to therapy based on CD47 blockade (107).

Antibiotics are widely used in the treatment of infectious diseases; however, their efficacy in cancer patients with co-existing infections remains highly controversial. Both retrospective and prospective cohort studies on NSCLC patients indicate that those receiving antibiotic treatment during PD-1/PD-L1 antibody therapy exhibit significantly reduced ORR, OS and PFS (72, 108, 109). Furthermore, broad-spectrum antibiotic therapy may increase tumor susceptibility by impairing specific immune surveillance mechanisms. For example, antibiotic-treated mice demonstrated significantly increased susceptibility to Lewis lung carcinoma due to defective induction of γδT17 cell responses (110). Nevertheless, this negative effect is not absolute. Evidence suggests that in NSCLC patients receiving first-line chemo-immunotherapy combination treatment, antibiotic exposure did not compromise therapeutic efficacy. This implies that antibiotics may exert complex, counterbalancing effects by simultaneously eliminating detrimental microbes and disrupting beneficial flora (111). This controversy largely stems from the broad-spectrum effects of systemic antibiotic administration, making it difficult for researchers to discern whether the immunotherapy outcomes result from the disruption of intratumoral microbiota or are a consequence of the antibiotic treatment itself (112).

Although numerous studies have confirmed a significant correlation between intratumoral microbiota and immunotherapy efficacy, the underlying molecular mechanisms remain incompletely understood. The latest research, based on real-world data from 16 cancer types, has developed the “IMIT” data platform. This platform integrates ORR stratification data with differences in intratumoral microbiota abundance to characterize the potential role of the intratumoral microbiome in cancer immunotherapy (113).

### 6 Modulating intratumoral microbiota to potentiate cancer therapy

Given the dual role of microbiota in cancer progression and therapy response, precise modulation strategies are urgently needed. Traditional antibiotic therapy for intratumoral microbial infections is significantly limited due to the complex nature of intratumoral microbial communities. Current therapeutic strategies are progressively shifting away from broad-spectrum eradication towards more targeted modulation. Recent advances in targeted nano-antibiotics offer a promising therapeutic alternative. Metronidazole and fluorouracil self-assemble into amphiphilic Metronidazole - Fluorouracil nanoparticles (MTI-FDU) in hydrophilic solutions (114). Within the glutathione (GSH)-enriched TME, specific cleavage of disulfide bonds linking metronidazole and fluorouracil enables MTI - FDU to selectively eliminate intratumoral bacteria and their byproducts, thereby reprogramming the immunosuppressive landscape (114). Capitalizing on the anaerobic activation properties of nitroimidazoles, researchers engineered a liposome-encapsulated silver-tinidazole antibiotic complex. This formulation specifically targets the pro-tumorigenic anaerobe *F. nucleatum*, inducing tumor-specific microbial neoantigen release while enhancing CD8^+^ T-cell infiltration (115). These studies demonstrate that nanocarrier technology offers a viable path for clinical translation by addressing the off-target effects associated with antibiotics.

Engineered probiotics, modified via synthetic biology and genetic engineering to carry specific components, can effectively repair or reconstruct the damaged TME. Genetically modified *Salmonella typhimurium*, engineered to stably express the pro-tumor-rejection cytokine LIGHT, demonstrates augmented oncolytic activity. This strain inhibits the progression and metastasis of lung cancer by concurrently inducing CD4^+^ and CD8^+^ T-cell immune responses (116). Genetically engineered bacterial outer membrane vesicles (OMVs) and hybrid membrane nanocarriers containing cytoplasmic membranes can simultaneously deliver multiple antigens and activate antigen - presenting cells. OMV-based carriers, via a surface plug-and- display system, rapidly present tumor antigens, suiting personalized cancer vaccines when neoantigens are identified. Hybrid carriers, combining surgical tumor cell membranes and bacterial cytoplasmic membranes, show advantages in presenting unknown neoantigens (117).

Oncolytic viruses (OVs), including both naturally occurring and genetically modified ones, can directly infect and preferentially lyse tumor cells. At the same time, they enhance the host’s antitumor immune response indirectly, making them promising candidates for precision cancer therapy (118). For example, the modified vaccinia virus TG4010, when used in combination with immunosuppressants, has been shown to increase the effectiveness of immunotherapy and improve PFS in NSCLC patients (119).

**Figure 5:**
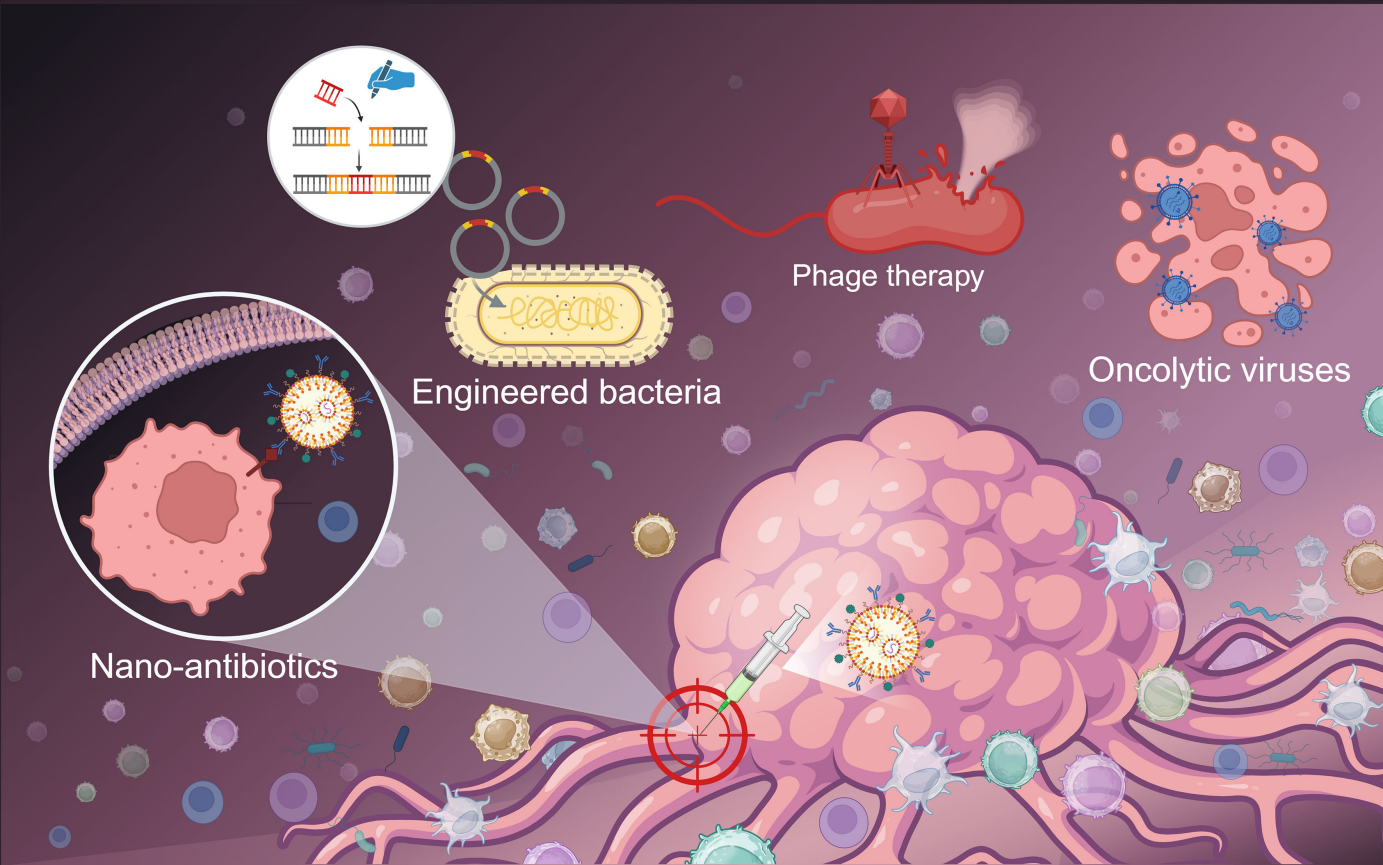
Modulating intratumoral microbiota to potentiate cancer therapy. Created in BioRender (2025). https://BioRender.com/e25wn1d.

Phages are gaining attention for their ability to specifically recognize and lyse bacteria (120). In oncology, azide-modified phages targeting *F. nucleatum* were engineered for covalent conjugate with irinotecan nanoparticles (121). This hybrid system significantly enhances the efficacy of first-line chemotherapy in malignant tumor (122). Furthermore, phage K and doxorubicin demonstrate synergistic activity against intracellular Staphylococcus aureus infections and metastasis in lung cancer chemotherapy models. This finding provides a mechanistic rationale for combining chemotherapeutics with phage therapy to combat intracellular pathogens (123). Although clinical trials directly applying phages to cancer treatment are limited, their programmable nature and microbial tropism establish phages as a promising approach for remodeling the tumor microecology (124) (**Figure 5**).

## 7 Intratumoral microbiota as potential biomarkers for lung cancer diagnosis and prognosis

Intratumoral microbiota and their metabolic factors show tumor-type and subtype specificity, differing significantly from those in healthy tissues. This makes them promising diagnostic biomarkers for cancer.

The BALF bacterial communities in patients with lung cancer are different from those with benign lesions. *Veillonella* and *Megasphaera* are more prevalent in lung cancer patients, showing potential as diagnostic biomarkers (125). Compared to healthy controls, *Streptococcus* is detected in lung cancer patients’ bronchial brush samples, having diagnostic and classification potential (126). However, while BALF and bronchial brushing samples can directly reflect the local microenvironment, they are limited by the invasiveness of the procedure and the risk of upper respiratory tract microbiota contamination.

In contrast, liquid biopsy offers higher clinical feasibility. Intratumoral microbial DNA is actively secreted via extracellular vesicles (EVs) or passively released due to tumor cell death into the TME. Subsequently, it enters the bloodstream, forming cmDNA (127). Based on whole-genome sequencing data of plasma from lung cancer patients and healthy controls, researchers have revealed significant differences in cmDNA characteristics associated with lung cancer. The cmDNA diagnostic model constructed from these features has shown excellent performance with high sensitivity in both lung cancer diagnosis and postoperative recurrence monitoring (128). However, due to the extremely low biomass of microbial DNA in the blood, this technique requires high sequencing depth and stringent contamination removal (129).

The distribution of intratumoral microbiota can serve as a novel biomarker for predicting survival and prognosis in lung cancer. In NSCLC patients, targeted sequencing of tumor samples and logistic regression analysis have identified four bacteria (*Haemophilus parainfluenzae*, *Serratia marcescens*, *Acinetobacter jungii*, and *Streptococcus constellation*) that can effectively predict the 2 - year survival rate (130). Another Research discovered that lung cancer patients with higher tumor tissue *Actinomycetales* and *Pseudomonadale* abundances have poorer DFS (131). Recently, researchers developed a clinical prognostic model based on 10 intratumoral microbial genera (such as *Sphingobacterium* and *Peptococcus*) associated with OS and PFS. This model accurately predicts a patient’s survival and recurrence status within 5 years, providing new biomarkers for prognostic stratification and management of NSCLC patients (67). In addition, *Peptococcus* has been discovered as a key independent prognostic factor for NSCLC, and its presence may indicate poor prognosis (67) (**Table 1**). These studies demonstrate that intratumoral microbiota hold considerable promise as prognostic markers, yet the discrepancies in results across different studies highlight the necessity for cross-cohort validation. Furthermore, the aforementioned research has not adequately adjusted for key confounding variables such as smoking history, prior antibiotic usage, and tumor staging. These environmental and clinical factors can profoundly reshape the pulmonary microbiota, potentially diminishing the reproducibility of microbial markers across diverse patient populations.

**Table 1:** Intratumoral microbiota as potential biomarkers for lung cancer diagnosis and prognosis.

Diagnosis/Prognosis	Microorganisms	Treatment method	Sample source	Clinical significance
Diagnostic biomarkers	*Veillonella and Megasphaera*	–	BALF	High - abundance *Veillonella* and Megasphaera as potential diagnostic biomarkers for lung cancer.
	*Streptococcus*	–	Bronchial specimen brushing samples	Diagnostic and classification potential.
Prognostic biomarkers	*F. Nucleatum*, Neisseria and *V. dispar*	Immune therapy	BALF	Significant association with immunotherapy resistance.
	*Gammaproteobacteria*	Immune therapy	Bronchoscopic tumor biopsy samples	Association with PD-L1 low expression and poor checkpoint immunotherapy response.
	*F. Nucleatum*	Immune therapy	Surgical or biopsy pathology	Associates with shorter OS/PFS in metastatic NSCLC.
	Escherichia	Monotherapy immunotherapy	Surgical or biopsy pathology	Association with Improved OS.
	*Haemophilus parainfluenzae*, *Serratia marcescens*, *Acinetobacter junii and Streptococcus constellation*	Targeted therapy or chemotherapy	Surgical or biopsy pathology	Predicts patients' 2-year survival rate.
	*Actinomycetales and* *Pseudomonadales*	Surgical treatment	Surgical pathology	Association with worse DFS.
	*Sphingobacterium, Peptococcus, Xanthobacter, Pantoea, Methylophilus, Oscillospira, Hydrogenispora, Woeseia, Pusillimonas and Glycomyces*	Surgical treatment	Surgical pathology	Accurately predicts 5-year survival/recurrence status.
	*Peptococcus*	Surgical treatment	Surgical pathology	Indicates poor prognosis.
	*Helicobacter pylori*	Immune therapy	Serum	Seropositivity correlates with reduced OS/PFS in anti-PD-1-therapy NSCLC.

Obtaining pathological tissue for prognosis prediction via surgery or biopsy is invasive and challenging, and using samples like sputum or BALF is prone to external microbial contamination (132). Liquid biopsies based on the microbiome show promise for improving cancer detection and prognosis. For instance, *Helicobacter pylori* seropositivity correlates with reduced survival in NSCLC patients on anti-PD-1 therapy, suggesting it as a prognostic serum biomarker (133). Crucially, a high intratumoral bacterial load with high iNOS expression implies a good prognosis, but a high bacterial load with increased FOXP3^+^ cells indicates a negative outcome, according to inflammatory infiltration analysis of the tumor stroma. This suggests that the local antitumor immune state determines the intratumoral microbiota’s prognostic significance (134). Integrating intratumoral bacterial load and immune marker expression can provide treatment guidance for patients with different risk profiles.

## 8 Limitations and challenges

### 8.1 Methodological challenges in low-biomass microbiome research

Unlike the microbiome-rich gut, the lung represents a low-biomass environment. This characteristic makes lung cancer microbiome research highly susceptible to external contamination.

Reagent contamination (Kitome) and batch effects are major threats in low-biomass studies. Due to the extremely low abundance of tumor-associated microbiomes, trace amounts of bacterial DNA from DNA extraction reagents, PCR systems, or laboratory environments can be erroneously amplified during sequencing, masking true biological signals. Studies have indicated that if stringent negative controls and contamination removal steps are not implemented, bacterial DNA contamination could lead to false-positive results (135). Additionally, using a single sequencing method and platform allows direct comparison of microbial diversity across samples from the same tumor type. However, variations in sample collection times and batch differences can directly influence the analysis results (13).

The preservation method of clinical samples significantly limits the accuracy of sequencing data. Formalin-fixed paraffin-embedded (FFPE) tissue is the most commonly used preservation method in clinical settings, but formalin fixation can cause chemical modifications, degradation, and fragmentation of DNA (136, 137). These chemical damages may result in sequencing errors and even misidentification of microbial species. Data indicate that in analyses of FFPE lung cancer samples, although the negative predictive value is high, the positive predictive value is only 80.0%, meaning that a significant portion of detected microbial signals could be due to technical errors (138). Furthermore, extended storage time and environmental contamination also limit FFPE samples’ ability to accurately reflect the true microbial community.

The heterogeneity of sampling sites and methods leads to low reproducibility across studies. Current data on the lung cancer microbiome are derived from a variety of sources, including BALF, protective specimen brushes (PSB), exhaled breath condensate (EBC), and surgically resected tissues. There are major differences in the detection protocols across studies, with each step potentially introducing specific technical biases (139). For example, EBC, as a non-invasive method, contains significantly less bacterial DNA than PSB samples, resulting in substantial differences in detection rates and diversity estimates (140, 141). Additionally, the lungs, as an open organ directly connected to the external environment via the upper respiratory tract, make microbiome sampling highly susceptible to contamination from bacteria in the mouth, throat, and nose.

Current detection methods suffer from insufficient resolution and lack of activity validation. Early studies largely relied on 16S rRNA gene sequencing, which typically only identifies bacteria at the genus level and lacks the resolution to differentiate at the strain level, limiting functional studies of specific pathogenic strains (132). More critically, DNA-based sequencing techniques cannot distinguish between live bacteria, dead bacteria, or cell-free DNA (142). Without verification through fluorescence *in situ* hybridization (FISH), bacterial culture, or RNA-level validation, it is difficult to confirm whether detected microorganisms are actively metabolizing, truly colonizing, or merely residual DNA.

Future lung cancer microbiome research must establish stringent standard operating procedures, including the introduction of rigorous blank controls to eliminate environmental errors. The use of fresh frozen tissues should be prioritized to reduce biases introduced by FFPE. Furthermore, integrating metagenomics with spatial visualization techniques (e.g., FISH) to visually and functionally validate the true presence and activity of microorganisms in the TME will help identify genuine tumor regulators.

### 8.2 Limitations in causality and experimental research models

Most current research on the intratumoral microbiota in lung cancer is based on cross-sectional studies, which primarily reveal correlations between microbiome abundance and lung cancer rather than establishing clear causal relationships. It remains unclear whether specific microorganisms actively drive the formation of an immunosuppressive microenvironment, or if the tumor’s hypoxic and necrotic environment passively selects and enriches these microorganisms.

There is a significant gap in evidence between human clinical samples, mouse models, and *in vitro* experiments. Existing molecular mechanism studies typically rely on *in vitro* cell co-culture or mouse models for validation. However, these approaches have notable limitations. *In vitro* experiments often lack the complexity of the TME (e.g., hypoxic conditions and matrix barriers), while the microbiome composition in experimental mice differs significantly from that of humans. Additionally, commonly used immune-deficient mice (such as those for xenograft models), which lack a full T-cell response, cannot accurately replicate the complex microbiome-immune dynamics in human lung cancer. These shortcomings in species differences and immune function make it difficult to directly translate findings from preclinical models to immune evasion phenomena observed in human patients.

### 8.3 Challenges in analyzing the non-bacterial microbial landscape of tumors

Current research on the lung cancer intratumoral microbiota is predominantly focused on bacteria, but the tumor microecology is, in fact, a multi-domain ecosystem composed of bacteria, fungi, viruses, and parasites. Research on the intratumoral microbiota has gradually expanded and made some progress, but the composition and function of viruses and parasites remain unclear, with research in these areas nearly nonexistent. This limits our comprehensive understanding of the immune remodeling mechanisms in the lung cancer microenvironment.

Microbiome analysis heavily relies on the completeness of reference genome databases. Unlike the well-established taxonomic databases for bacterial 16S rRNA, the reference genome databases for fungi, viruses, and parasites are fragmented and inadequately annotated (143, 144). This leads to a significant number of sequencing results that cannot be accurately mapped to specific species, severely hindering the accurate identification and tracing of non-bacterial microorganisms. Even if viral or parasitic sequences are detected through sequencing, distinguishing whether they represent latent infections or true oncogenic drivers remains a challenge. For example, microbes such as EBV or CMV are commonly latent in healthy individuals, and detection at the DNA level alone cannot prove their transcriptional activity or pathogenic causality in tumors. To address this, transcriptomic analysis of gene expression activity is needed, but this increases the requirements for sample quality.

Tumor samples may harbor multiple microbial communities, but current extraction methods struggle to simultaneously capture multiple microbial domains, limiting the in-depth exploration of intratumoral microbiota interactions. However, understanding these cross-domain microbial interactions is crucial. For instance, CMV infection has been shown to enhance bacterial adhesion and invasiveness on the cell surface, and the two synergistically influence the malignant behavior of lung cancer cells (145). Additionally, co-infection by parasites and viruses has demonstrated significant carcinogenic synergy, where co-infection substantially increases cancer risk compared to single-pathogen infections. Parasite infections can promote viral cell proliferation and induce immune-related gene expression, thereby accelerating tumor progression (146). Only by overcoming these technical barriers can we truly decode the full ecological landscape of the lung cancer intratumoral microbiota and develop novel combination immunotherapies targeting the synergistic effects of specific pathogens.

### 8.4 Challenges in clinical translation and application of intratumoral microbiota

Although manipulating the intratumoral microbiota to enhance cancer treatment shows promising therapeutic potential, translating this approach into standard clinical practice still faces significant challenges related to safety and feasibility. For example, while engineered *Salmonella typhimurium* have been designed to deliver cytokines such as LIGHT to activate antitumor immunity, their biosafety remains a primary obstacle for clinical translation. Advanced lung cancer patients are often severely immunocompromised. The lung is a highly vascularized organ that is highly sensitive to inflammatory responses. Therefore, even attenuated bacterial strains administered there risk triggering systemic sepsis or an uncontrolled cytokine storm. Moreover, the clinical use of OVs primarily involves intravenous injection, which results in the dilution of the virus in peripheral circulation. The virus may also be prematurely cleared by antibodies or complement in the bloodstream, potentially reducing the effective viral dose reaching the tumor site. Therefore, further evaluation of the pharmacokinetics, dosing, and treatment regimens for OVs is necessary.

The clinical application of intratumoral microbiota as diagnostic and prognostic biomarkers in lung cancer faces dual challenges: insufficient specificity and incompatibility with existing workflows. While studies have identified potential diagnostic biomarkers such as *Veillonella*, the diagnostic efficacy (sensitivity and specificity) of microbiome-based biomarkers remains uncertain when compared to clinical diagnostic “gold standards” such as low-dose spiral CT or histopathology. Furthermore, the feasibility of integrating microbiome testing into clinical workflows is limited. Current microbiome analysis relies on high-throughput sequencing and complex bioinformatics interpretation, which are incompatible with standardized procedures commonly used in hospital pathology departments, such as IHC or PCR. Sequencing is time-consuming, expensive, and requires strict sample collection and contamination control, which makes it challenging to meet clinical demands for rapid diagnosis and cost-effective screening. Therefore, for intratumoral microbiota to transition from the laboratory to clinical practice, standardized clinical microbiome testing procedures need to be established. These procedures should demonstrate, through validation in large multicenter cohorts, that they offer superior or complementary diagnostic value compared to existing methods.

## 9 Conclusion

The intratumoral microbiota in lung cancer may contribute to reshaping the TME and influencing malignant progression by triggering a synergistic network of chronic inflammation and immunosuppression. On one hand, microbial components and their toxins activate inflammatory signaling pathways, promoting the release of pro-inflammatory factors such as IL-1β, IL-17, and TNF-α, which help recruit neutrophils, Th17 cells, and other cells that maintain a pro-tumor inflammatory environment. On the other hand, intratumoral microbiota and their metabolites recruit immunosuppressive cells such as Tregs, M2 macrophages, and MDSCs, upregulate PD-L1 expression, and suppress T/NK cell activity, thereby forming an immune evasion barrier. This inflammation-immunity imbalance is further amplified by the direct effects of microbes on cancer cells, collectively driving lung cancer progression and resistance to immunotherapy. Crucially, this microbe-tumor interaction demonstrates clear biological plausibility. It is underpinned by the lung’s unique anatomical status as a mucosal organ interacting with the external environment, facilitating microbial entry via barrier disruption and vascular leakiness. Once established, the tumor’s characteristic hypoxia, nutrient abundance, and immune-privileged nature provide a selective sanctuary, fostering a pathological symbiosis that drives disease progression and therapeutic resistance.

While advancements in science and technology have facilitated breakthroughs in intratumoral microbiota research, this field still faces several limitations. Current studies predominantly focus on bacteria and fungi, with insufficient attention given to other components such as intratumoral viruses and parasites. Furthermore, the low-biomass nature of the lung renders sequencing results highly vulnerable to contamination from reagents and the environment, underscoring an urgent need to establish standardized protocols for sample processing and bioinformatic decontamination. Future research must move beyond single-species analysis. It should employ multi-omics technologies to comprehensively decipher multi-kingdom microbial networks. Moreover, while ensuring biosafety, efforts should advance towards clinical trials of engineered microbial therapies. The ultimate goal is to realize precision diagnosis and treatment for lung cancer by targeting the microbial microenvironment.
